# Synthesis, structure and solvatochromic properties of some novel 5-arylazo-6-hydroxy-4-phenyl-3-cyano-2-pyridone dyes

**DOI:** 10.1186/1752-153X-6-71

**Published:** 2012-07-23

**Authors:** Adel Alimmari, Dušan Mijin, Radovan Vukićević, Bojan Božić, Nataša Valentić, Vesna Vitnik, Željko Vitnik, Gordana Ušćumlić

**Affiliations:** 1 Department of Organic Chemistry, Faculty of Technology and Metallurgy, University of Belgrade, Karnegijeva 4, P.O. Box 3503, 11120, Belgrade, Serbia; 2 Department of Chemistry, ICTM, University of Belgrade, Studentski trg 12-16, 11000, Belgrade, Serbia; 3 Faculty of Chemistry, University of Belgrade, Studentski trg 12-16, 11000, Belgrade, Serbia

**Keywords:** Arylazo pyridone dyes, Tautomerism, Solvent effect, Substituent effect, DFT caculation

## Abstract

**Background:**

A series of some novel arylazo pyridone dyes was synthesized from the corresponding diazonium salt and 6-hydroxy-4-phenyl-3-cyano-2-pyridone using a classical reaction for the synthesis of the azo compounds.

**Results:**

The structure of the dyes was confirmed by UV-vis, FT-IR, ^1^H NMR and ^13^C NMR spectroscopic techniques and elemental analysis. The solvatochromic behavior of the dyes was evaluated with respect to their visible absorption properties in various solvents.

**Conclusions:**

The azo-hydrazone tautomeric equilibration was found to depend on the substituents as well as on the solvent. The geometry data of the investigated dyes were obtained using DFT quantum-chemical calculations. The obtained calculational results are in very good agreement with the experimental data.

## Background

Azo dyes are the most widely used compounds in various fields, such as the dyeing of textiles, in biological-medical studies and advanced applications in organic synthesis [1-4]. The success of azo colorants is due to the simplicity of their synthesis by diazotization and azo coupling, the almost innumerable possibilities presented by variation of the diazo compound and coupling component, the generally high molar extinction coefficient and the medium to high light and wet fastness properties [5]. In recent years, azo dyes have gained wide interest and found many uses in materials for optical applications and in analysis. Due to their properties, including optical storage capacity, optical switching, holography and non-linear optical properties, polymers with azo units represent promising candidates for photoactive materials [6]. Pyridone derivatives are heterocyclic intermediates relatively recently employed for the preparation of arylazo dyes and several investigations on substituted arylazo pyridones were performed and reviewed [7-12]. The physico-chemical properties of these dyes are closely related to their tautomerism. Determination of azo-hydrazone tautomers in both the solid state and solution phase is quite interesting from both the theoretical and practical standpoints, since the tautomers have different technical properties and dyeing performances. In previous publications, the absorption spectra of ten 5-arylazo-6-hydroxy-4-methyl-3-cyano-2-pyridones in different solvents were studied and the results showed that these dyes exist in the hydrazone tautomeric form in the solid state and in the solvent DMSO-d_6_ while equilibriums exist between the hydrazone and the azo form in different solvents [13].

**Scheme 1:**
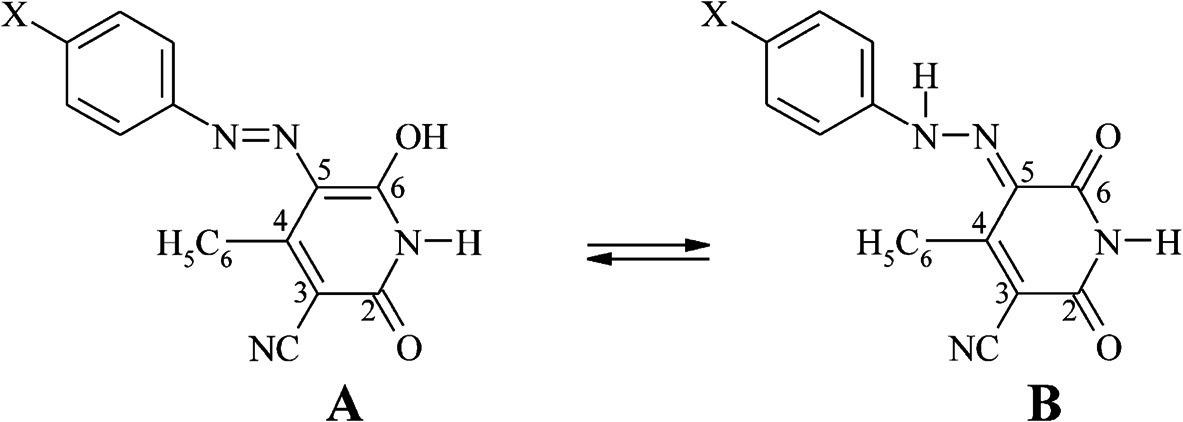
**The equilibrium between the azo form (A) and the hydrazone form (B) of 5-arylazo-6-hydroxy-4-phenyl-3-cyano-2-pyridones.** (X = H (**1**), OH (**2**), OCH_3_ (**3**), CH_3_ (**4**), Cl (**5**), Br (**6**), I (**7**), F(**8**), CN (**9**), COOH (**10**), COCH_3_ (**11**), NO_2_ (**12**)).

In this work, the synthesis of twelve new 5-arylazo-6-hydroxy-4-phenyl-3-cyano-2-pyridones (Scheme 1), their UV-vis absorption spectra (200–600 nm) in thirteen solvents of different polarity and the relationship between color and the constitution of these dyes are reported. The effects of the solvent and the substituent on the azo-hydrazone tautomeric equilibrium, and the geometry data of the investigated dyes, obtained using DFT quantum-chemical calculations, were studied and evaluated.

## Experimental

### General

All starting materials were obtained from Aldrich and Fluka, and were used without further purification.

The IR spectra were determined using a Bomem MB-Series Fourier Transform-infrared (FT-IR) spectrophotometer in the form of KBr pellets. The ^13^C and ^1^H NMR spectral measurements were performed on a Varian Gemini 2000 (200 MHz). The spectra were recorded at room temperature in deuterated dimethyl sulfoxide (DMSO-d_6_). The chemical shifts are expressed in ppm values referenced to TMS. The ultraviolet-visible (UV-vis) absorption spectra were recorded on a Schimadzu 1700 spectrophotometer in the region 200–600 nm. The spectra were run in spectroquality solvents (Fluka) using concentration of 1 × 10^–5^ M. All melting points were uncorrected and are in degree Celsius. Elemental analysis was performed using a VARIO EL III elemental analyzer. Characterization data are given in Additional files 1, 2, 3, 4, 5, 6, 7.

### Preparation of 5-arylazo-6-hydroxy-4-phenyl-3-cyano-2-pyridone dyes (1–12)

All the investigated arylazo pyridone dyes were synthesized from the corresponding diazonium salts and 4-phenyl-6-hydroxy-3-cyano-2-pyridone using a classical reaction for the synthesis of azo compounds [5]. 4-Phenyl-6-hydroxy-3-cyano-2-pyridone was prepared from ethyl benzoylacetate and cyanoacetamide using a modified literature procedure [14].

The yields of the dyes were in the range 50–75%.

## Results and discussion

### Spectral characteristics and tautomerism

The arylazo pyridone dyes prepared in this work may exist in two main tautomeric forms (Scheme 1). Generally, tautomers not only have different colors, but also have different tinctorial strength and different properties, *e.g*., light fastness [7]. Due to the commercial importance of arylazo pyridone dyes, the azo-hydrazone tautomerism has been intensively studied [15-17]. It was concluded that the equilibrium between the two tautomers is influenced by the structure of the compound and the solvent used [15-17].

The infrared spectra of all the synthesized dyes showed two intense carbonyl bands at about 1630 and 1684 cm^–1^, which were assigned to the diketohydrazone form. The FT-IR spectra also showed a band at 3110–3217 cm^–1^, assigned to the imino group (N–H) of the heterocyclic (pyridone) ring and a band at 3382–3414 cm^–1^ that was assigned to the N–H of hydrazo tautomeric form.

The ^1^H NMR spectra of the dyes exhibited a broad signal near 14.27–14.93 ppm. This signal corresponds to the imine N–H proton resonance of the hydrazone form (Scheme 1, Structure **B**). In our previous publication [13], the ^1^H NMR spectra of ten 5-arylazo-6-hydroxy-4-methyl-3-cyano-2-pyridones were studied and the results showed that these dyes existed in the hydrazone tautomeric form in the solid state and in the solvent DMSO-d_6_, with N–H peaks in the range 14.35–14.87 ppm.

N. Ertan *et al*. [10] reported the ^1^H NMR spectra of some azo pyridone dyes in CF_3_COOD / CDCl_3_ and showed that these dyes existed in the hydrazone form with the N–H peak in the range 15.10–15.60 ppm. Q. Peng *et al*. [5,15] also reported the ^1^H NMR spectra of azo pyridone dyes in CDCl_3_ and concluded that the azo pyridone dyes exist in the hydrazone form and with the N–H peaks appearing within the range 14.30–16.09 ppm.

Lucka and Machacek [18] and Cee *et al*. [19] concluded from ^13^C NMR studies of some N-alkyl derivatives of azopyridones that pyridone azo dyes in CDCl_3_ and DMSO-d_6_ exist in the hydrazone form. Our results are in agreement with these results.

### Solvent effects

**Figure 1:**
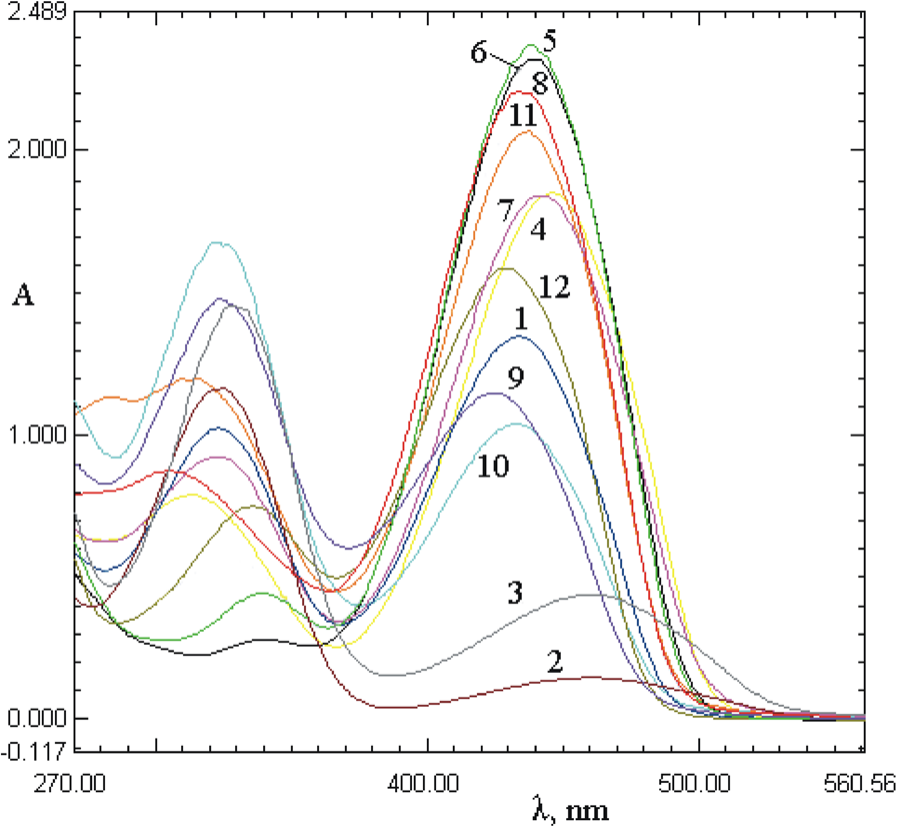
Absorption spectra of dyes 1–12 in methanol.

**Figure 2:**
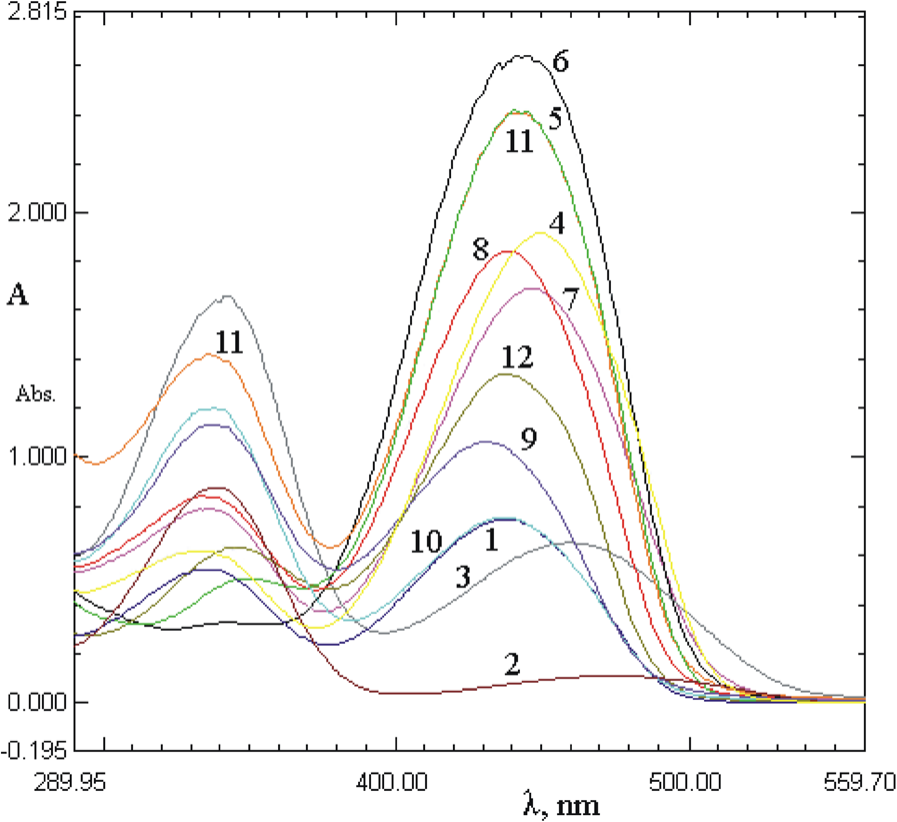
Absorption spectra of dyes 1–12 in dimethyl sulfoxide.

Since the tautomeric equilibria strongly depend on the nature of the media, the behavior of selected arylazo pyridone dyes in thirteen protic and aprotic solvents was studied. For this purpose, the absorption spectra of the pyridone dyes (**1**–**12**) at a concentration 1 × 10^–5^ mol dm^–3^ were recorded over the *λ* range between 200 and 600 nm in the selected solvent set. The characteristic absorption spectra of the investigated azo dyes in methanol and dimethyl sulfoxide are shown in Figures 1 and 2.

The UV-vis absorption spectra of all the dyes showed a weak band at about 260–370 nm, assigned to the azo tautomeric form and a strong band at 375–550 nm, which was assigned to hydrazone tautomeric form. The absorption maxima, which correspond to a transition in which electron density is transferred from the hydrazone –NH group to the pyridone carbonyl group (lower energy band), are presented in Tables 1 and 2. It was observed that, although slightly positive solvatochromism is evident, the absorption spectra of dyes **1**–**12** did not change significantly in all the employed solvents and the absorption maxima did not correlate with the polarity of the solvent.

**Table 1:** Absorption maxima of the hydrazone tautomer (B) of arylazo pyridone dyes (1–12) in protic solvents

**Dye No.**	***λ***_**max**_**(nm)**					
	**Methanol**	**Ethanol**	**Propan-1-ol**	**Propan-2-ol**	**Butan-1-ol**	**2-Methylpropan-2-ol**
**1**	433	434	434	433	433	431
**2**	460	470	476	472	470	474
**3**	460	458	458	455	448	454
**4**	445	446	447	445	446	445
**5**	437	435	438	439	439	439
**6**	437	438	438	438	442	440
**7**	440	442	443	442	442	442
**8**	433	436	436	434	436	436
**9**	424	426	425	425	425	424
**10**	432	432	433	433	433	433
**11**	437	436	436	435	435	435
**12**	429	428	429	428	428	427

**Table 2:** Absorption maxima of hydrazone tautomer (B) of arylazo pyridone dyes (1–12) in aprotic solvents

**Dye No.**	***λ***_**max**_**(nm)**						
	**Tetrahydrofuran**	**Dioxane**	**Methyl acetate**	**Ethyl acetate**	**Dimethylformamide**	**Dimethylacetamide**	**Dimethyl sulfoxide**
**1**	431	430	430	429	415	428	437
**2**	466	461	460	460	470	470	473
**3**	455	455	454	454	449	456	459
**4**	442	442	441	440	423	441	449
**5**	434	437	434	433	417	427	440
**6**	436	437	434	433	421	425	441
**7**	439	441	437	437	425	432	447
**8**	432	432	431	429	410	422	437
**9**	426	426	423	423	433	434	430
**10**	431	432	429	429	431	433	437
**11**	434	436	433	432	436	439	442
**12**	429	430	427	427	451	452	437

**Figure 3:**
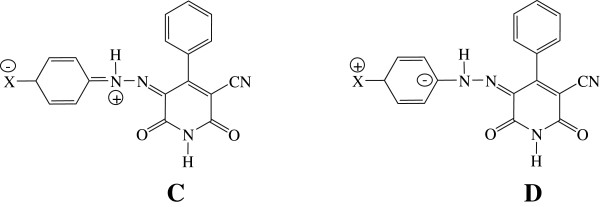
Resonance effect of electron-accepting (structure C) and electron-donating (structure D) substituents of the arylazo component on the hydrazone tautomer (B).

Additional evidence for the solvent effect on the structure-property relationship of arylazo pyridone dyes was obtained from the correlation of the absorption frequencies (*ν* = 1 / *λ* in cm^–1^) for the hydrazone tautomeric form (Tables 1 and 2) with the Kamlet-Taft Solvatochromic Equation (1) [20] of the following form:

(1) $$\;\nu =\nu_{0}\text{+ }s\pi *+b\beta +a\alpha$$

where π* is an index of the solvent dipolarity / polarizability, *β* is a measure of the solvent hydrogen-bonding acceptor (HBA) basicity, *α* is a measure of the solvent hydrogen-bonding donor (HBD) acidity and *ν*_0_ is the regression value of the solute property in cyclohexane as the reference solvent. The regression coefficients *s*, *b* and *a* in Eq. 1 are a measure the relative susceptibilities of the absorption frequencies to the indicated solvent parameters. The linear solvation energy relationship (LSER) concept developed by Kamlet and Taft is one of the most ambitions and successful quantitative treatments of solvation effects. This treatment assumes attractive interactions between a solute and its environment and enables an estimation of the ability of the investigated compounds to form hydrogen bonds. The solvent parameters [21] are given in Table 3. The correlations of the absorption frequencies *ν*_max_ for hydrazone tautomer were realized by means of multiple linear regression analysis. It was found that *ν*_max_ in the selected solvent sets showed satisfactory correlation with the π*, *β* and *α* parameters. The results of the multiple regressions are presented in Tables 4 and 5, and the coefficients *ν*_0_, *s*, *b* and *a* (Table 4) fitted at the 95% confidence level. The negative sign of the *a* coefficient (Table 4) for all dyes (excluding the H, CN, COOH and NO_2_ substituents) and the *s* and *b* coefficients for strong electron-donating substituents and strong electron-accepting substituents indicate a bathochromic shifts with increasing solvent dipolarity / polarizability and solvent hydrogen bond acidity and basicity. This suggests stabilization of the electron excited state relative to the ground state. The positive sign of the *a* coefficient for strong electron-accepting substituents and the *s* and *b* coefficients for moderate electron-donating and electron-accepting substituents indicate hypsochromic shifts with increasing solvent dipolarity / polarizability and both types of hydrogen bonding effects. These results showed that the solvent effect on the UV-vis absorption spectra of the investigated azo pyridone dyes is very complex and strongly dependent on the nature of the substituent on the arylazo component. They also indicated that the electronic behavior of the nitrogen atoms of hydrazone group are somewhat different between derivatives with electron-donating and electron-accepting substituents (Figure 3, Structures **C** and **D**). This phenomenon is caused by the difference in the conjugational or migrating ability of the electron lone pairs on the nitrogen atoms of the pyridone azo dyes. The strong electron-donating substituents in the phenyl group produce extensive delocalization in the arylazo group (Figure 3, Structure **D**), while the influence of the strong electron-accepting substituents are opposite, due to the positive charge on the nitrogen atom in the hydrazone tautomer (Figure 3, Structure **C**).

**Table 3:** **Solvent parameters**[21]

**No.**	**Solvent**	π*	***β***	***α***
1	Methanol	0.60	0.62	0.93
2	Ethanol	0.54	0.77	0.83
3	Propan-1-ol	0.52	0.83	0.8
4	Propan-2-ol	0.48	0.95	0.76
5	Butan-1-ol	0.47	0.88	0.79
6	2-Methylpropan-2-ol	0.41	0.11	0.68
7	Tetrahydrofuran	0.58	0.55	0
8	Dioxane	0.55	0.37	0
9	Methyl acetate	0.60	0.42	0
10	Ethyl acetate	0.55	0.45	0
11	*N*,*N*-Dimethylformamide	0.88	0.69	0
12	*N*,*N*-Dimethylacetamide	0.88	0.76	0
13	Dimethyl sulfoxide	1.00	0.76	0

**Table 4:** **Regression fits to the solvatochromic parameters (Eq.1)**

**No.**	**Substituent**	***ν***_**0 **_**(10**^**3**^**cm**^**–1**^**)**	***s *** **(10**^**3**^**cm**^**–1**^**)**	***b *** **(10**^**3**^**cm**^**–1**^**)**	***a *** **(10**^**3**^**cm**^**–1**^**)**	***r***^**a**^	***s***^**b**^	***F***^**c**^	**Solvents used in the calculation**^**d**^
**1**	H	21.92	1.81	0.80	0.52	0.9789	0.078	46	1–11
		(±0.180)	(±0.240)	(±0.170)	(±0.100)				
**2**	OH	21.34	−0.53	−0.86	−0.20	0.9417	0.099	21	2–13
		(±0.155)	(±0.269)	(±0.280)	(±0.191)				
**3**	OCH_3_	23.02	−0.66	0.22	−0.31	0.9614	0.037	24	1–10,13
		(±0.159)	(±0.089)	(±0.078)	(±0.104)				
**4**	CH_3_	23.02	−0.44	−0.19	−0.24	0.8128	0.099	5	1–10,12,13
		(±0.159)	(±0.222)	(±0.197)	(±0.118)				
**5**	Cl	21.28	2.57	0.61	−0.31	0.9802	0.081	57	1–11
		(±0.183)	(±0.246)	(±0.176)	(±0.098)				
**6**	Br	21.61	2.11	0.38	−0.27	0.9703	0.085	38	1–11
		(±0.194)	(±0.260)	(±0.186)	(±0.104)				
**7**	I	21.39	2.01	0.50	−0.25	0.9812	0.067	52	1–9,11
		(±0.164)	(±0.210)	(±0.147)	(±0.083)				
**8**	F	21.14	3.02	0.79	−0.43	0.9738	0.113	43	1–11
		(±0.256)	(±0.344)	(±0.246)	(±0.138)				
**9**	CN	24.50	−1.21	−0.51	0.10	0.9862	0.042	71	1–7,10–12
		(±0.102)	(±0.114)	(±0.097)	(±0.051)				
**10**	COOH	24.58	−1.25	−0.57	0.09	0.9823	0.046	64	1–7, 9–12
		(±0.097)	(±0.121)	(±0.098)	(±0.055)				
**11**	COCH_3_	23.73	−0.92	−0.23	−0.16	0.9878	0.027	94	1−12
		(±0.050)	(±0.062)	(±0.057)	(±0.033)				
**12**	NO_2_	27.02	−5.14	−1.71	0.47	0.9735	0.176	42	1–7,9,10,12,13
		(±0.398)	(±0.533)	(±0.382)	(±0.214)				

^a^ Correlation coefficient.

^b^ Standard error of the estimate.

^c^ Fisher's test.

^d^ Solvent number as given in Table 3.

**Table 5:** Percentage contribution of the solvatochromic parameters

**Substituent**	***P***_π*_(%)	***P***_**β**_**(%)**	***P***_**α**_**(%)**
H	58	25	17
OH	33	54	13
OCH_3_	55	19	26
CH_3_	50	22	28
Cl	74	17	9
Br	76	14	40
I	73	18	9
F	71	19	10
CN	66	28	6
COOH	65	30	5
COCH_3_	70	18	12
NO_2_	70	23	7

The percentage contributions of the solvatochromic parameters (Table 5) for the azo dyes with strong and moderate electron-accepting substituents on the arylazo group, showed that the most of the solvatochromism is due to the solvent dipolarity / polarizability rather than to the solvent acidity and basicity. These results could be explained by the effect of the positive charge on the nitrogen atom in the hydrazone tautomer (Figure 3, Structure **C**) and stabilization of this form mostly due to the solvent dipolarity / polarizability (non-specific solute-solvent interactions) than by hydrogen bond donating and hydrogen bond accepting properties (specific solute-solvent interactions).

### Substituent effects

As seen in Tables 1 and 2, the absorption spectra of the *p*-nitro derivative (dye **12**) were shifted hypsochromically in all used solvents (excluding DMSO and DMF) when compared with dye **1**. Moreover, the absorption spectra of the *p*-hydroxy and *p*-methoxy derivatives (dyes **2** and **3**) were shifted bathochromically in all used solvents when compared with dye **1**. It is well known that the *λ*_max_ values of the hydrazone tautomeric form of an azo dyes will show a general shift to shorter wavelengths when substituents of increasing electron withdrawing strength are introduced into the ring of the diazo component. In contrast, electron donor substituents produce strong bathochromic shifts [2]. The results presented in Tables 1 and 2 are in agreement with these conclusions. Thus, the observed relationship between the substituent constants and the *λ*_max_ values strongly suggests that the lower energy absorption maxima of the investigated azo dyes originate from hydrazones (Scheme 1, Structure **B**).

**Figure 4:**
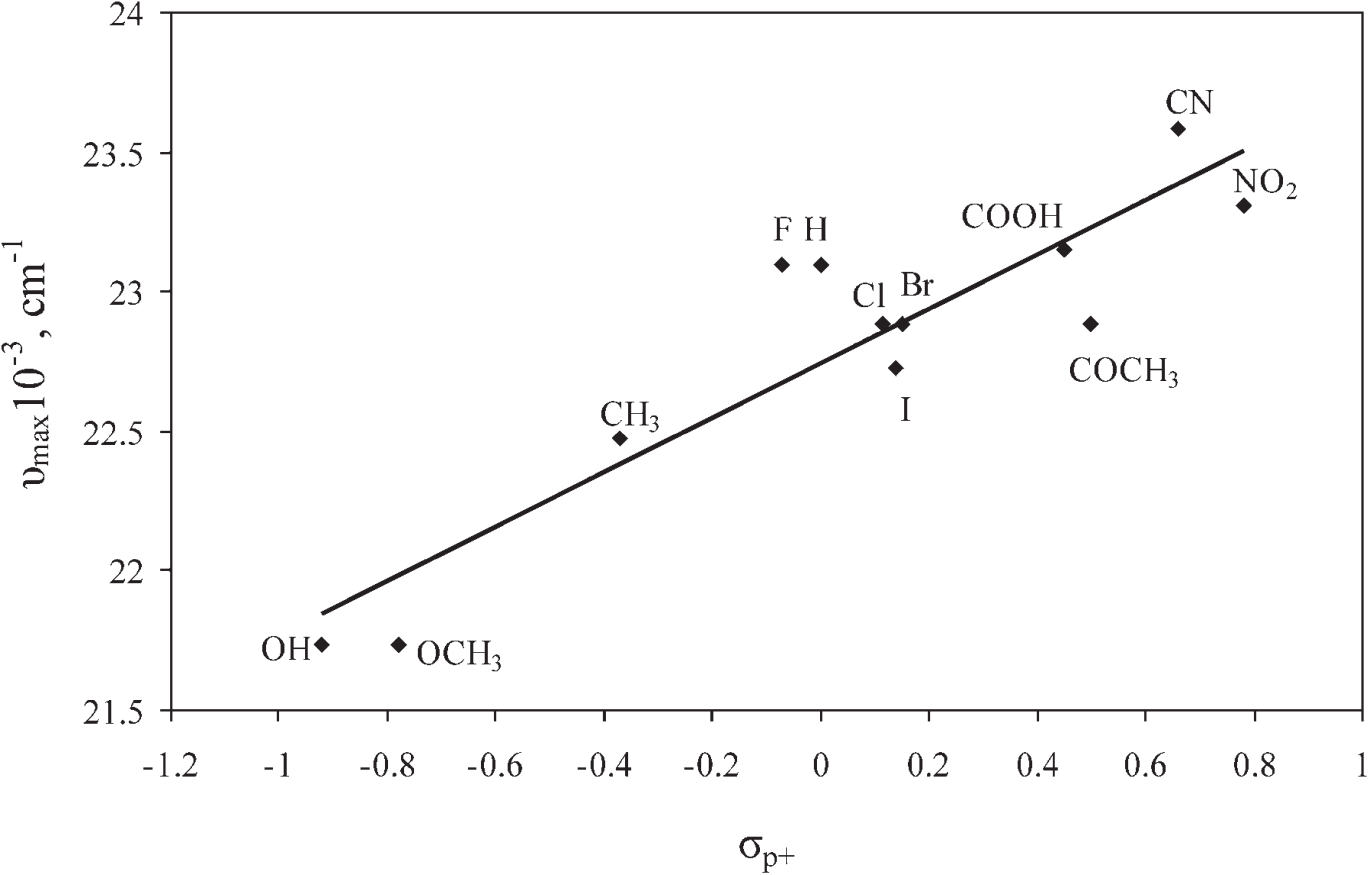
**Relationship between *ν***_**max **_**and** *** σ***_**p+ **_**for arylazo pyridone dyes 1–12 in methanol.**

In order to explain these results, the absorption frequencies were correlated by the Hammett Equation (2) using *σ*_p_ or *σ*_p+_ substituent constants [22]:

(2) $$\;\nu =\nu_{0}\text{+ }\rho \sigma_{\text{p}}$$

where *ρ* is a proportionality constant reflecting the sensitivity of the absorption frequencies to the substituent effects. The substituent *σ*_p_ or *σ*_p*+*_ constants measure the electronic effect of the substituents. The plot *ν*_max_*vs*. the *σ*_p_ substituent constants gave a correlation which showed deviations from the Hammet Equation in all dipolar aprotic solvents. However, a linear Hammett correlation was obtained in protic solvents. A better correlation of *ν*_max_ was obtained with the *σ*_p*+*_ substituent constants [23] than with the *σ*_p_ constants in all solvents, which indicates extensive delocalization in the arylazo group. The existence of the linear correlation with positive slope presented in Figure 4 and Equation 3 was interpreted as evidence of the diketohydrazone structure.

(3) $$\;\nu_{\text{max}}=0.974\sigma_{\text{p}+}+\;22.744$$

(6) $$\;\left(r=0.9114,s=0.25,F=49,n=\;12\right)$$

### Quantum chemical calculations

**Figure 5:**
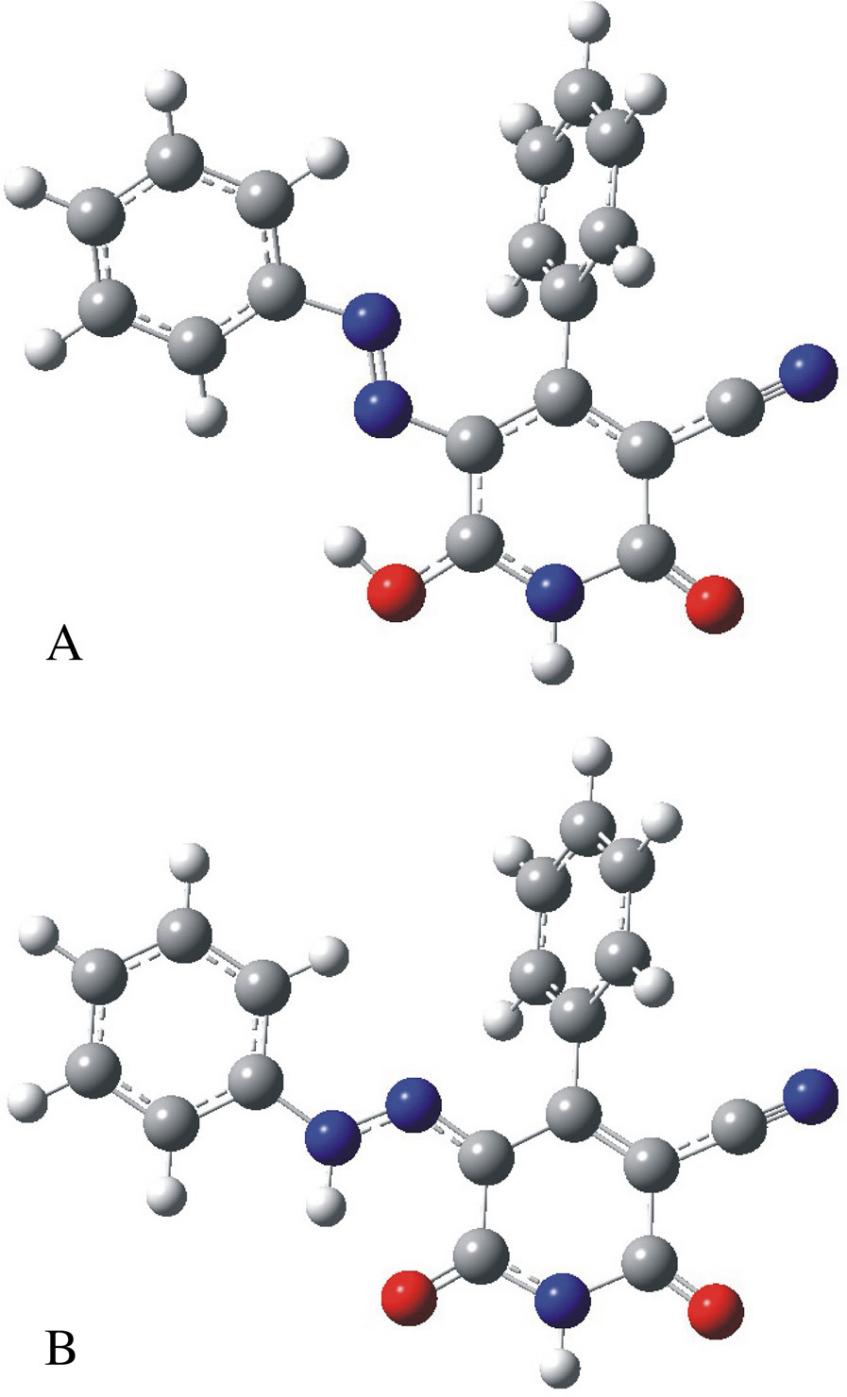
The most stable azo (A) and hydrazone (B) tautomer optimized structures of dye 1, at the B3LYP/6-31G(d) level of theory.

DFT calculations were performed for different azo-hydrazone tautomers of dye **1**. The structures were preliminary optimized by the semi-empirical PM3 method and the most stable geometries in vacuum were re-optimized at the B3LYP/6-31G(d) level of theory [24,25]. The Gaussian 03 program package was used [26]. The DFT calculations suggested that the diketohydrazone form (**B**) (Figure 5) is the most stabile tautomer of dye **1**. The relative energies and the statistical Boltzmann distribution weighted values of the most stable azo-hydrazone tautomers of dye **1** are given in Table 6.

**Table 6:** **The relative energies**^**a **^**and the statistical Boltzmann distribution weighted values of the most stable azo-hydrazone tautomers of dye 1**

**Tautomer of dye 1**	**Relative energies [kcal mol**^**–1**^**]**	**Statistical Boltzmann distribution weighted values [%]**
**A**	21.729	0.00
**B**	0.000	100.00

^a^ The energies were calculated relative to the energy of the most stable tautomer **(B**) of dye **1**, which represents an energy minimum (Data were obtained at the B3LYP/6-31G* level).

## Conclusions

In this work, twelve new 5-arylazo-6-hydroxy-4-phenyl-3-cyano-2-pyridone dyes were synthesized. Characterization and the absorption ability of the dyes were studied. The results showed that the solvent effect on UV-vis absorption spectra of the investigated arylazo pyridone dyes is very complex and strongly depends on the nature of the substituent on the arylazo component. The introduction of electron-donating substituents into the arylazo ring results in strong bathochromic shifts in all solvents. These solvatochromic properties are evident for the hydrazone tautomeric form. The introduction of electron-accepting substituents into the arylazo moiety produces slight bathochromic or hypsochromic effects. The satisfactory correlation of the ultraviolet absorption frequencies of the hydrazone tautomeric form of the azo dyes with Eq. (1) indicates that the correct model was selected. It was demonstrated that a solvatochromic equation with three solvatochromic parameters π*, *β* and *α* can be used to evaluate the effects of both types of hydrogen bonding and of the solvent dipolarity / polarizability effect. All the synthesized dyes exist in the hydrazone tautomeric form in the solid state and dyes were predominantly as hydrazones in all the employed solvents. The calculational results of the geometry data of the investigated dyes, obtained using DFT quantum-chemical calculations, were in very good agreement with the experimental data.

## Competing interests

The authors declare that they have no competing interests.

## Authors’ contributions

AA, BB and RV performed the synthesis and characterization of the compounds. NV, ŽV and VV realized the calculations of the molecular descriptors and performed the statistical analysis. DM and GU conceived the study and participated in its design and coordination. All authors read and approved of the final manuscript.

## Supplementary Material

Additional file 1 **Experimental details and data of the investigated compounds.** Additional file 1 includes the experimental procedures and the results of the physico–chemical characterization of the investigated compounds [5,14]. Click here for file

Additional file 2 ^1^H NMR (200 MHz, DMSO-*d_6_*) spectra of 5-(4-bromophenylazo)-6-hydroxy-4-phenyl-3-cyano-2-pyridoine (6). Click here for file

Additional file 3 ^13^C NMR (50 MHz, DMSO-*d_6_*) spectra of 5-(4-bromophenylazo)-6-hydroxy-4-phenyl-3-cyano-2-pyridoine (6). Click here for file

Additional file 4 IR spectra of 5-(4-bromophenylazo)-6-hydroxy-4-phenyl-3-cyano-2-pyridoine (6). Click here for file

Additional file 5 ^1^H NMR (200 MHz, DMSO-*d_6_*) spectra of 5-(4-nitrophenylazo)-6-hydroxy-4-phenyl-3-cyano-2-pyridoine (12). Click here for file

Additional file 6 ^13^C NMR (50 MHz, DMSO-*d_6_*) spectra of 5-(4-nitrophenylazo)-6-hydroxy-4-phenyl-3-cyano-2-pyridoine (12). Click here for file

Additional file 7 IR spectra of 5-(4-nitrophenylazo)-6-hydroxy-4-phenyl-3-cyano-2-pyridoine (12). Click here for file
