# Supplementary material for: Synthesis, structure and solvatochromic properties of some novel 5-arylazo-6-hydroxy-4-phenyl-3-cyano-2-pyridone dyes
Source: Chem Cent J. 2012 Jul 23;6:71. doi: 10.1186/1752-153X-6-71 (PMC3537587; doi:10.1186/1752-153X-6-71)
Supplement: Additional file 1 — Experimental details and data of the investigated compounds. Additional file 1 includes the experimental procedures and the results of the physico–chemical characterization of the investigated compounds [5,14]. [file 1752-153X-6-71-S1.doc]

**(Additional file 1 - Experimental details and data of the investigated compounds)**

**Synthesis, structure and solvatochromic properties of some novel 5-arylazo-6-hydoxy-4-phenyl-3-cyano-2-pyridone dyes**

Adel Alimmari^a^, Dušan Mijin^a^,*, Radovan Vukićević^a^, Bojan Božić^a^, Nataša Valentić^a^, Vesna Vitnik^b^, Željko Vitnik^c^, Gordana Ušćumlić^a^

^a^ Department of Organic Chemistry, Faculty of Technology and Metallurgy, University of Belgrade, Karnegijeva 4, P.O. Box 3503, 11120 Belgrade, Serbia

^b^ Department of Chemistry, IChTM, University of Belgrade, Studentski trg 12–16, 11000 Belgrade, Serbia

^c^ Faculty of Chemistry, University of Belgrade, Studentski trg 12–16, 11000 Belgrade, Serbia

^*^Corresponding author:

Tel.: +381 11 3303671

fax: +381 11 3370387

E-mail address:

AA: [adelarbi@hotmail.com](mailto:ADELARBI@hotmail.com)

DM: [kavur@tmf.bg.ac.rs](mailto:kavur@tmf.bg.ac.rs)

RV: [raks_vuk@yahoo.com](mailto:raks_vuk@yahoo.com)

BB: [bbozic84@yahoo.com](mailto:bbozic84@yahoo.com)

NV: [naca@tmf.bg.ac.rs](mailto:naca@tmf.bg.ac.rs)

VV: [vesnak@chem.bg.ac.rs](mailto:vesnak@chem.bg.ac.rs)

ŽV: [zvitnik@chem.bg.ac.rs](mailto:zvitnik@chem.bg.ac.rs)

GU: [goca@tmf.bg.ac.rs](mailto:goca@tmf.bg.ac.rs)

**General Procedure for the Preparation of 4-Phenyl-6-hydroxy-3-cyano-2-pyridone**

4-Phenyl-6-hydroxy-3-cyano-2-pyridone was prepared from ethyl benzoylacetate and cyanoacetamide using a modified literature procedure [1]. Equimolar amounts (10 mmol) of ethyl benzoylacetate and cyanoacetamide were heated under reflux in absolute ethanol (10 mL) in the presence of potassium hydroxide (10 mmol) as catalyst for 20 h. The product was isolated by filtration and purified by crystallization from ethanol.

*4-Phenyl-6-hydroxy-3-cyano-2-pyridone*. White crystalline solid; m.p.: 279–280 °C (lit. m.p.: 280 °C [1]); yield: 62 %; IR (KBr, ν / cm^-1^): 3419 (OH), 3321 (NH), 2227 (CN), 1654 (C=O); ^1^H NMR (200 MHz, DMSO-*d*_6_, δ / ppm): 6.26 (1H, s, PyrH); 7.60–7.40 (5H, m, PhH); 11.74 (1H, s, OH).

**General Procedure for the Preparation of 5-arylazo-6-hydoxy-4-phenyl-3-cyano-2-pyridone dyes (1–12)**

All the investigated arylazo pyridone dyes were synthesized from the corresponding diazonium salts and 4-phenyl-6-hydroxy-3-cyano-2-pyridone using classical reaction for the synthesis of the azo compounds [2]. The obtained compounds were purified by crystallization from acetone and then analyzed.

*5-phenylazo-6-hydroxy-4-phenyl-3-cyano-2-pyridoine* (**1**). Orange crystalline solid; m.p.: 257–260 °C; yield: 75 %; anal. calcd. for C_18_H_12_N_4_O_2_: C, 68.35; H, 3.82; N, 17.71; found: C, 68.51; H, 3.75; N, 17.57; IR (KBr, ν / cm^-1^): 3390 (NH of hydrazone form), 3153 (NH on heterocyclic), 2229 (CN), 1654, 1630 (C=O on heterocyclic); ^1^H NMR (200 MHz, DMSO-*d*_6_, δ / ppm): 7.42–7.10 (5H, m, ArH); 7.70–7.42 (5H, m, ArH); 12.22 (1H, s, NH on heterocyclic); 14.61 (1H, s, NH of hydrazone form); ^13^C NMR (50 MHz, DMSO-*d*_6_, δ / ppm): 163.24 (C2), 96.80 (C3), 162.02 (C4), 117.26 (C5), 161.29 (C6).

*5-(4-hydroxyphenylazo)-6-hydroxy-4-phenyl-3-cyano-2-pyridoine* (**2**). Dark red crystalline solid; m.p.: 272–274 °C; yield: 58 %; anal. calcd. for C_18_H_12_N_4_O_3_: C, 65.06; H, 3.64; N, 16.86; found: C, 65.22; H, 3.72; N, 16.72; IR (KBr, ν / cm^-1^): 3385 (NH of hydrazone form), 3153 (NH on heterocyclic), 2230 (CN), 1654, 1637 (C=O on heterocyclic); ^1^H NMR (200 MHz, DMSO-*d*_6_, δ / ppm): 7.27 (2H, d, *J* = 9 Hz, ArH); 7.60–7.40 (5H, m, ArH); 8.77 (2H, d, *J* = 9.6 Hz, ArH); 10.04 (1H, s, OH substituent); 12.12 (1H, s, NH on heterocyclic); 14.93 (1H, s, NH of hydrazone form); ^13^C NMR (50 MHz, DMSO-*d*_6_, δ / ppm): 163.40 (C2), 97.04 (C3), 162.26 (C4), 118.98 (C5), 161.53 (C6).

*5-(4-methoxyphenylazo)-6-hydroxy-4-phenyl-3-cyano-2-pyridoine* (**3**). Brown crystalline solid; m.p.: 268–270 °C; yield: 60 %; anal. calcd. for C_19_H_14_N_4_O_3_: C, 65.89; H, 4.07; N, 16.18; found: C, 66.06; H, 3.96; N, 16.72; IR (KBr, ν / cm^-1^): 3386 (NH of hydrazone form), 3152 (NH on heterocyclic), 2231 (CN), 1664, 1642 (C=O on heterocyclic); ^1^H NMR (200 MHz, DMSO-*d*_6_, δ / ppm): 3.72 (3H, s, OCH_3_ substituent); 7.60–7.45 (5H, m, ArH); 7.75–7.60 (4H, m, ArH); 12.15 (1H, s, NH on heterocyclic); 14.82 (1H, s, NH of hydrazone form); ^13^C NMR (50 MHz, DMSO-*d*_6_, δ / ppm): 163.31 (C2), 96.87 (C3), 162.11 (C4), 119.05 (C5), 161.44 (C6).

*5-(4-methylphenylazo)-6-hydroxy-4-phenyl-3-cyano-2-pyridoine* (**4**). Red crystalline solid; m.p.: 263–267 °C; yield: 54 %; anal. calcd. for C_19_H_14_N_4_O_2_: C, 69.08; H, 4.27; N, 16.96; found: C, 69.24; H, 4.13; N, 16.02; IR (KBr, ν / cm^-1^): 3387 (NH of hydrazone form), 3135 (NH on heterocyclic), 2224 (CN), 1661, 1640 (C=O on heterocyclic); ^1^H NMR (200 MHz, DMSO-*d*_6_, δ / ppm): 2.30 (3H, s, CH_3_ substituent); 7.60–7.42 (7H, m, ArH); 7.66 (2H, d, *J* = 9.6 Hz, ArH); 12.19 (1H, s, NH on heterocyclic); 14.68 (1H, s, NH of hydrazone form); ^13^C NMR (50 MHz, DMSO-*d*_6_, δ / ppm): 163.28 (C2), 96.82 (C3), 162.04 (C4), 117.27 (C5), 161.31 (C6).

*5-(4-chlorophenylazo)-6-hydroxy-4-phenyl-3-cyano-2-pyridoine* (**5**). Light orange crystalline solid; m.p.: 272–274 °C; yield: 72 %; anal. calcd. for C_18_H_11_ClN_4_O_2_: C, 61.64; H, 3.16; N, 15.97; found: C, 61.48; H, 3.08; N, 15.76; IR (KBr, ν / cm^-1^): 3406 (NH of hydrazone form), 3110 (NH on heterocyclic), 2217 (CN), 1660, 1631 (C=O on heterocyclic); ^1^H NMR (200 MHz, DMSO-*d*_6_, δ / ppm): 7.26 (2H, d, *J* = 9, ArH); 7.38 (2H, d, *J* = 8.4 Hz, ArH); 7.70–7.44 (5H, m, ArH); 12.26 (1H, s, NH on heterocyclic); 14.52 (1H, s, NH of hydrazone form); ^13^C NMR (50 MHz, DMSO-*d*_6_, δ / ppm): 162.97 (C2), 96.80 (C3), 161.82 (C4), 118.77 (C5), 161.20 (C6).

*5-(4-bromophenylazo)-6-hydroxy-4-phenyl-3-cyano-2-pyridoine* (**6**). Yellow crystalline solid; m.p.: 269–271 °C; yield: 68 %; anal. calcd. for C_18_H_11_BrN_4_O_2_: C, 54.70; H, 2.81; N, 14.18; found: C, 54.82; H, 2.72; N, 14.06; IR (KBr, ν / cm^-1^): 3439 (NH of hydrazone form), 3170 (NH on heterocyclic), 2224 (CN), 1697, 1651 (C=O on heterocyclic); ^1^H NMR (200 MHz, DMSO-*d*_6_, δ / ppm): 7.16 (2H, d, *J* = 8.4, ArH); 7.70–7.44 (7H, m, ArH); 12.26 (1H, s, NH on heterocyclic); 14.48 (1H, s, NH of hydrazone form); ^13^C NMR (50 MHz, DMSO-*d*_6_, δ / ppm): 161.82 (C2), 101.07 (C3), 161.19 (C4), 119.20 (C5), 160.95 (C6).

*5-(4-iodophenylazo)-6-hydroxy-4-phenyl-3-cyano-2-pyridoine* (**7**). Dark red crystalline solid; m.p.: 267–270 °C; yield: 52 %; anal. calcd. for C_18_H_11_IN_4_O_2_: C, 48.89; H, 2.51; N, 12.67; found: C, 48.66; H, 2.38; N, 12.48; IR (KBr, ν / cm^-1^): 3398 (NH of hydrazone form), 3217 (NH on heterocyclic), 2225 (CN), 1684, 1651 (C=O on heterocyclic); ^1^H NMR (200 MHz, DMSO-*d*_6_, δ / ppm): 7.04 (2H, d, *J* = 9, ArH); 7.70–7.44 (5H, m, ArH); 7.67 (2H, d, *J* = 8.4 Hz, ArH); 12.24 (1H, s, NH on heterocyclic); 14.49 (1H, s, NH of hydrazone form); ^13^C NMR (50 MHz, DMSO-*d*_6_, δ / ppm): 163.24 (C2), 96.80 (C3), 161.89 (C4), 119.23 (C5), 161.24 (C6).

*5-(4-fluorophenylazo)-6-hydroxy-4-phenyl-3-cyano-2-pyridoine* (**8**). Brown crystalline solid; m.p.: 262–264 °C; yield: 50 %; anal. calcd. for C_18_H_11_FN_4_O_2_: C, 64.67; H, 3.32; N, 16.76; found: C, 64.42; H, 3.24; N, 16.58; IR (KBr, ν / cm^-1^): 3385 (NH of hydrazone form), 3153 (NH on heterocyclic), 2224 (CN), 1654, 1628 (C=O on heterocyclic); ^1^H NMR (200 MHz, DMSO-*d*_6_, δ / ppm): 7.20 (2H, d, *J* = 9, ArH); 7.62–7.40 (5H, m, ArH); 7.69 (2H, d, *J* = 8.4 Hz, ArH); 12.22 (1H, s, NH on heterocyclic); 14.59 (1H, s, NH of hydrazone form); ^13^C NMR (50 MHz, DMSO-*d*_6_, δ / ppm): 163.27 (C2), 96.85 (C3), 161.89 (C4), 119.28 (C5), 161.15 (C6).

*5-(4-cyanophenylazo)-6-hydroxy-4-phenyl-3-cyano-2-pyridoine* (**9**). Dark red crystalline solid; m.p.: 270–272 °C; yield: 48 %; anal. calcd. for C_19_H_11_N_5_O_2_: C, 66.86; H, 3.25; N, 20.52; found: C, 66.71; H, 3.18; N, 20.38; IR (KBr, ν / cm^-1^): 3386 (NH of hydrazone form), 3152 (NH on heterocyclic), 2231 (CN), 1668, 1652 (C=O on heterocyclic); ^1^H NMR (200 MHz, DMSO-*d*_6_, δ / ppm): 8.03–7.40 (9H, m, ArH); 12.33 (1H, s, NH on heterocyclic); 14.29 (1H, s, NH of hydrazone form); ^13^C NMR (50 MHz, DMSO-*d*_6_, δ / ppm): 163.28 (C2), 96.85 (C3), 161.70 (C4), 117.48 (C5), 161.13 (C6).

*5-(4-carboxyphenylazo)-6-hydroxy-4-phenyl-3-cyano-2-pyridoine* (**10**). Dark red crystalline solid; m.p.: 260–262 °C; yield: 54 %; anal. calcd. for C_19_H_12_N_4_O_4_: C, 63.33; H, 3.36; N, 15.55; found: C, 63.52; H, 3.22; N, 15.38; IR (KBr, ν / cm^-1^): 3382 (NH of hydrazone form), 3168 (NH on heterocyclic), 2231 (CN), 1683, 1654 (C=O on heterocyclic); ^1^H NMR (200 MHz, DMSO-*d*_6_, δ / ppm): 7.60–7.40 (7H, m, ArH); 7.66 (2H, d, *J* = 9.6 Hz, ArH); 12.30 (1H, s, NH on heterocyclic); 14.27 (1H, s, NH of hydrazone form); ^13^C NMR (50 MHz, DMSO-*d*_6_, δ / ppm): 163.33 (C2), 96.83 (C3), 161.95 (C4), 116.96 (C5), 161.24 (C6).

*5-(4-acetylphenylazo)-6-hydroxy-4-phenyl-3-cyano-2-pyridoine* (**11**). Dark red crystalline solid; m.p.: 264–266 °C; yield: 58 %; anal. calcd. for C_20_H_14_N_4_O_3_: C, 67.03; H, 3.94; N, 15.63; found: C, 67.16; H, 3.68; N, 15.47; IR (KBr, ν / cm^-1^): 3385 (NH of hydrazone form), 3145 (NH on heterocyclic), 2231 (CN), 1667, 1635 (C=O on heterocyclic); ^1^H NMR (200 MHz, DMSO-*d*_6_, δ / ppm): 2.50 (3H, s, CH_3_CO substituent); 7.32 (2H, d, *J* = 8.4 Hz, ArH); 7.65–7.43 (5H, m, ArH); 7.90 (2H, d, *J* = 8.6 Hz, ArH); 12.34 (1H, s, NH on heterocyclic); 14.46 (1H, s, NH of hydrazone form); ^13^C NMR (50 MHz, DMSO-*d*_6_, δ / ppm): 163.27 (C2), 96.84 (C3), 161.80 (C4), 116.88 (C5), 161.22 (C6).

*5-(4-nitrophenylazo)-6-hydroxy-4-phenyl-3-cyano-2-pyridoine* (**12**). Dark yellow crystalline solid; m.p.: 268–270 °C; yield: 60 %; anal. calcd. for C_18_H_11_N_5_O_4_: C, 59.84; H, 3.07; N, 19.38; found: C, 59.97; H, 2.98; N, 19.21; IR (KBr, ν / cm^-1^): 3429 (NH of hydrazone form), 3148 (NH on heterocyclic), 2227 (CN), 1671, 1646 (C=O on heterocyclic); ^1^H NMR (200 MHz, DMSO-*d*_6_, δ / ppm): 7.40 (2H, d, *J* = 9, ArH); 7.65–7.45 (5H, m, ArH); 8.19 (2H, d, *J* = 9 Hz, ArH); 12.37 (1H, s, NH on heterocyclic); 14.37 (1H, s, NH of hydrazone form); ^13^C NMR (50 MHz, DMSO-*d*_6_, δ / ppm): 161.58 (C2), 103.14 (C3), 161.09 (C4), 117.30 (C5), 160.78 (C6).

**References**

1. Roch J, Müller E, Narr B, Nickl J, Haarmann W: **3-Amino-4-phenyl-6-piperidino-1H-pyrazolo[3,4-b]-pyridines and salts thereof**. US 4260621, 1981
2. Chen C, Wang I: **Synthesis of some pyridone azo dyes from 1-substitued 2-hydroxy-6-pyridone derivatives and their colour assessment**. Dyes Pigm 1991, **15**:69–82.
